# Dand5 is involved in zebrafish tailbud cell movement

**DOI:** 10.3389/fcell.2022.989615

**Published:** 2023-01-09

**Authors:** Catarina Bota, Gabriel G. Martins, Susana S. Lopes

**Affiliations:** ^1^ iNOVA4Health, NOVA Medical School Faculdade de Ciências Médicas, NMS|FCM, Universidade Nova de Lisboa, Lisboa, Portugal; ^2^ Instituto Gulbenkian de Ciência, Fundação Calouste Gulbenkian, Oeiras, Portugal

**Keywords:** Dand5, pre-somitic mesoderm, somites, asymmetry, symmetry, left-right development, cell migration

## Abstract

During vertebrate development, symmetry breaking occurs in the left-right organizer (LRO). The transfer of asymmetric molecular information to the lateral plate mesoderm is essential for the precise patterning of asymmetric internal organs, such as the heart. However, at the same developmental time, it is crucial to maintain symmetry at the somite level for correct musculature and vertebrae specification. We demonstrate how left-right signals affect the behavior of zebrafish somite cell precursors by using live imaging and fate mapping studies in dand5 homozygous mutants compared to wildtype embryos. We describe a population of cells in the vicinity of the LRO, named Non-KV Sox17:GFP+ Tailbud Cells (NKSTCs), which migrate anteriorly and contribute to future somites. We show that NKSTCs originate in a cluster of cells aligned with the midline, posterior to the LRO, and leave that cluster in a left-right alternating manner, primarily from the left side. Fate mapping revealed that more NKSTCs integrated somites on the left side of the embryo. We then abolished the asymmetric cues from the LRO using dand5−/− mutant embryos and verified that NKSTCs no longer displayed asymmetric patterns. Cell exit from the posterior cluster became bilaterally synchronous in dand5−/− mutants. Our study revealed a new link between somite specification and Dand5 function. The gene dand5 is well known as the first asymmetric gene involved in vertebrate LR development. This study revealed a new link for Dand5 as a player in cell exit from the maturation zone into the presomitic mesoderm, affecting the expression patterns of myogenic factors and tail size.

## Introduction

Visceral organs exhibit Left-Right (LR) asymmetry regarding position and pattern in vertebrates, despite showing a bilaterally symmetric body plan. Generally, symmetry breaking occurs at an organ called the Left-Right Organizer (LRO), where in most vertebrates, polarized cilia produce an asymmetric fluid flow leading to the degradation of *dand5 mRNA* on the left side (Marques et al., 2004; Lopes et al., 2010; Schweickert et al., 2010). Such mRNA degradation was recently shown to be mediated by Bicc1 and Dicer (Maerker et al., 2021; Minegishi et al., 2021). As Dand5 is a Nodal inhibitor, Nodal persists only on the left side of the lateral plate mesoderm (LPM) and establishes an asymmetric cascade of gene expression. The subsequent activation of the transcription factor Pitx2 on the left LPM leads to asymmetric organogenesis (extensively reviewed in Grimes and Burdine, 2017; Schweickert et al., 2017; Zinski et al., 2017).

In zebrafish, the LRO or Kupffer’s vesicle (KV) is a transient fluid-filled, monociliated spheroid structure that develops during the early segmentation period at the posterior end of the notochord (Amack and Yost, 2004; Essner, 2005). KV is derived from a cluster of superficial cells close to the embryonic shield, known as dorsal forerunner cells (DFCs) (Cooper and Amico, 1996). The expression of *dand5* in the KV is initially symmetric and around 8 ss, it becomes asymmetric in a fluid flow-dependent manner, biased to the right side (Lopes et al., 2010). Motile cilia in the KV generate counterclockwise fluid flow (Essner, 2005; Supatto et al., 2008), which is highly regulated (Sampaio et al., 2014; Tavares et al., 2017) and is perceived by the polycystin complex consisting of *pkd1l1* and *pkd2* (Pennekamp et al., 2002; Schottenfeld et al., 2007; Field et al., 2011; Kamura et al., 2011; Yoshiba et al., 2012; Yuan et al., 2015) in either mechanosensing or chemosensing unknown processes.

*Southpaw (spaw)* is the zebrafish nodal gene involved in the establishment and transduction of LR asymmetry*.* It is first expressed in a symmetric bilateral shape in cells near the KV at 4–6 ss (Long et al., 2003). Nodal proteins form complexes with type II and type I Activin receptors serine/threonine kinases, to activate the nodal pathway and target promoters (reviewed in Schier, 2003; Shen, 2007). Thus, later at 10–12 ss, *spaw* expression becomes asymmetrical in the left LPM (Long et al., 2003) where Southpaw will induce its own mRNA expression spreading from the posterior to the anterior range of the left LPM until 22s (Wang and Yost, 2008)**.** The left-sided confinement of Spaw is achieved by the presence of different secreted factors from the TGF-β family: a midline barrier conferred by Lefty1 (Wang and Yost, 2008)**,** an anterior barrier due to the presence of Lefty2 in the left cardiac field (Long et al., 2003) and a posterior barrier by BMP signaling (Lenhart et al., 2011).

Breaking symmetry is crucial for correct internal organ placement, and to produce symmetrical somites, thereby laying the foundation for symmetrical musculature, vertebrae, and innervation. Somites form in a symmetrical periodic fashion from the unsegmented presomitic mesoderm (PSM) through genetic oscillations of a segmentation clock (Oates et al., 2012). It was previously reported that somitic symmetry is achieved by actively shielding the PSM from LR signals from the LRO and LPM (Kawakami et al., 2005; Vermot and Pourquié, 2005). A recent study reported that zebrafish somites form with asymmetric differences in length and position, which are then corrected by somite surface tension (Naganathan et al., 2022). Somite precursors come from a dorsal tailbud population of neuromesodermal progenitor cells (Kanki and Ho, 1997; Martin and Kimelman, 2012) that commit to mesoderm fate and then move ventrally to the maturation zone (MZ) where cells become migratory (Kanki and Ho 1997; Griffin and Kimelman 2002; Fior et al., 2012; Manning and Kimelman 2015). MZ cells then generate cell flows towards the PSM, where cell motility decreases to form somites (Kanki and Ho, 1997; Lawton et al., 2013; Banavar et al., 2021).

In zebrafish, many studies have been performed to track cell movement, focusing either on posterior elongation (Lawton et al., 2013; Mongera et al., 2018; Das et al., 2019; Banavar et al., 2021) or the tailbud exit of somite precursors (Fior et al., 2012; O’Neill and Thorpe, 2013; Manning and Kimelman, 2015; Goto et al., 2017). However, whether somite precursor cells display LR asymmetries in their first movements toward the PSM or even when forming somites *in vivo* has never been reported (Kawakami et al., 2005).

To address these questions, we took advantage of the inadvertent labeling of a mesoderm tailbud population using a Tg(sox17:GFP) line and followed cell behaviors by live imaging over several hours. We identified a group of cells that displayed asymmetries by acquiring successive anterior positions on the left side of the embryo. In addition, we found that such asymmetries started earlier, with an asymmetric cell exit from a cluster of cells posterior to the KV in the MZ region. Then, by fate mapping, we demonstrated that cells displaying asymmetric behaviors are somite precursors and integrate somites with a left bias alternating manner in wildtype (WT) embryos that is lost in *dand5* ^*−/−*^ embryos. In summary, we describe somite precursors displaying asymmetric behaviors *in vivo* in response to LR signals that are lost in the absence of *dand5*.

## Materials and methods

### Zebrafish lines and embryo manipulation

The zebrafish strains used in this study were WT (AB strain—ZFIN), transgenic Tg (sox17:GFP)^*s870Tg*^ (Sakaguchi et al., 2006), Tg (act2b:LIFEACT-RFP) ^*e115Tg*^ (Behrndt et al., 2012) and mutant *dand5* ^*a204/a204*^ (Montague et al., 2018). Adult zebrafish were kept in a recirculating water treatment unit system at 28.0°C, pH 7, and 950 μS/m conductivity under a 10 h dark/14 h light cycle. They were spawned by pair mating, and eggs were collected and staged according to standard protocols (Kimmel et al., 1995; Dahm et al., 2002). Embryos were raised in E3 medium (5 mM NaCl, 0.17 mM KCl, 0.33 mM CaCl2, 0.33 mM MgSO4, with 0.0001% methylene blue at early stages) at 28.0°C.

### Embryo microinjections

For Kaede photoconversion, 100 pg of *Kaede* mRNA were injected into one-cell stage WT and Tg (sox17:GFP) embryos.

### Immunostaining and *in situ* hybridization

Whole-mount immunostaining was performed as described previously (Lopes et al., 2010) using the following antibodies: anti-MF20 (1:100; DSHB), anti-fibronectin (1:100; Sigma), anti-GFP (1:500; Invitrogen), Alexa Fluor 488 (Invitrogen; 1:500), Alexa Fluor 546 (Invitrogen; 1:500), and Alexa Fluor 647 (Invitrogen; 1:500). Flat-mounted 13–16 ss embryos and 24 hpf embryos mounted in 1% low-melting agarose (LMA) with the dorsal side facing the glass bottom of the dish were imaged using a Zeiss LSM 980 Airyscan confocal microscope. Individual sox17, *myoD*, double *myoD*, and *dand5* whole-mount *in situ* hybridization was performed as previously described (Thisse and Thisse, 2014). The embryos were photographed using a Zeiss Axio Imager Z2 upright microscope. Gene expression for *myoD* and double staining for *myoD* and *dand5* were scored blindly for the different genotypes and analyzed by the chi-square test using Prism 8 (GraphPad).

### Live imaging and photoconversion

For live imaging of sox17:GFP^+^ cells in whole zebrafish embryos, 10 ss embryos were mounted in 1% LMA (low melting agarose) in a Petri dish with the dorsal side facing the objective and fully submerged in E3. Images of the KV and adjacent cells were acquired using a Praire Ultima two-photon system mounted on an Olympus BX60 upright microscope. Z-stacks were obtained at 2 μm intervals at 28°C and time cycles of 3 and 6 min for 3–6 h 10 ss *dand5* ^*−/−*^ embryos were mounted in 1% LMA, with the tail facing the bottom of the dish, and images were acquired using a Zeiss LSM 980 Airyscan confocal microscope. 10 ss *Tg(sox17:GFP);Tg(act2b:LIFEACT-RFP)* double-transgenic embryos were mounted in 1% LMA, with the tail facing the bottom of the dish, and images were acquired using a Zeiss LSM 980 Airyscan confocal microscope. Z-stacks were acquired at 0.35 µm step size at 1 s intervals. Z-stacks were obtained at 2 μm intervals at 28°C for 2 min for 60–90 min. Kaede photoconversion was performed using a Leica TCS SP5 confocal laser scanning microscope with the FRAP module. *Kaede* mRNA-injected 8 ss embryos were mounted in 1% LMA and the targeted cluster of sox17:GFP^+^ cells localized posterior to the KV was subjected to a 9 s exposure to a 50% 405 nm UV laser. Images of the entire tail and targeted region were acquired before, during, and post-UV exposure. After photoconversion, the embryos were released into the E3 medium at 28°C until 14 ss or 24 hpf. 14 ss embryos were fixed in 4% PFA ON, and 24 hpf embryos were mounted in 1% LMA in a dorsal position for tail z-stack acquisition using a two-photon microscope.

### Body length measurement

To measure body length, 3 days post-fertilization (dpf), WT (AB), and *dand5* ^*−/−*^ larvae were mounted in 2% methylcellulose and photographed using a Zeiss Axio Observer. Larvae were measured using the FIJI software.

### Imaging of fixed embryos

For imaging of sox17:GFP + cells, WT 10 ss, WT, and *dand5* ^*−/−*^ 13 and 14 ss embryos were fixed in 4% PFA and stored in PBS at 4°C. To preserve uniformity between analyses, embryos were mounted in 1% LMA at the same position as described here for live imaging. Z-stacks were acquired on a Praire Ultima two-photon system at 1 μm intervals. Kaede photoconverted WT 14 ss embryos were imaged using a Zeiss LSM 980 Airyscan confocal microscope.

To evaluate KV morphology, 8 ss WT and *dand5* ^*−/−*^ embryos raised in the sox17:GFP background were fixed in 4% PFA ON, and z-stacks of the KV were acquired using a Zeiss LSM 980 Airyscan confocal microscope.

### Image analysis

Datasets were first processed using the FIJI/ImageJ software (Schindelin et al., 2012). Imaris v9.8.1 (Bitplane) was used to render Z-stacks of live-imaged sox17:GFP^+^ cells and produce accelerated 3D movies from which we manually tracked NKSTC cells (on both the left and right sides) and the KV centroid. The number of tracks and spots, anterior position (µm), anterior track distance (µm), the shortest distance to KV (µm), the shortest distance to nearest neighbor (µm), and track speed (µm/s) were computed with Imaris v9.8.1 (Bitplane) and extracted. KV 3D surfaces were acquired by manual drawing in every slice, where KV cells were always visible when the KV was still open. For fixed embryos, z-stacks of sox17:GFP^+^ cells were reconstructed in 3D using Imaris v9.8.1 (Bitplane), and x, y, and z coordinates for Left and Right cells plus the center point of the lumen of the KV were extracted. These coordinates were then imported into a MATLAB 2019a script written to calculate the number of cells on both the left and right sides, the distance of cells to the KV lumen (µm), and the anterior distance of cells to the KV (µm) (or y distance) as a measure of the displacement along the Anterior-Posterior (AP) axis of the embryos. The number of Kaede-photoconverted cells was scored using the FIJI software. The somite height and width were measured in FIJI. The morphology of the KV lumen was assessed by 3D projections using IMARIS v9.8.1 (Bitplane) and manually tracing the KV lumen to produce representative 3D surfaces. The calculated volume (µm^3^), area (µm^2^), and sphericity were extracted. All paired and unpaired comparisons were performed using the *t*-test in Prism 8 (GraphPad).

## Results

**FIGURE 1:**
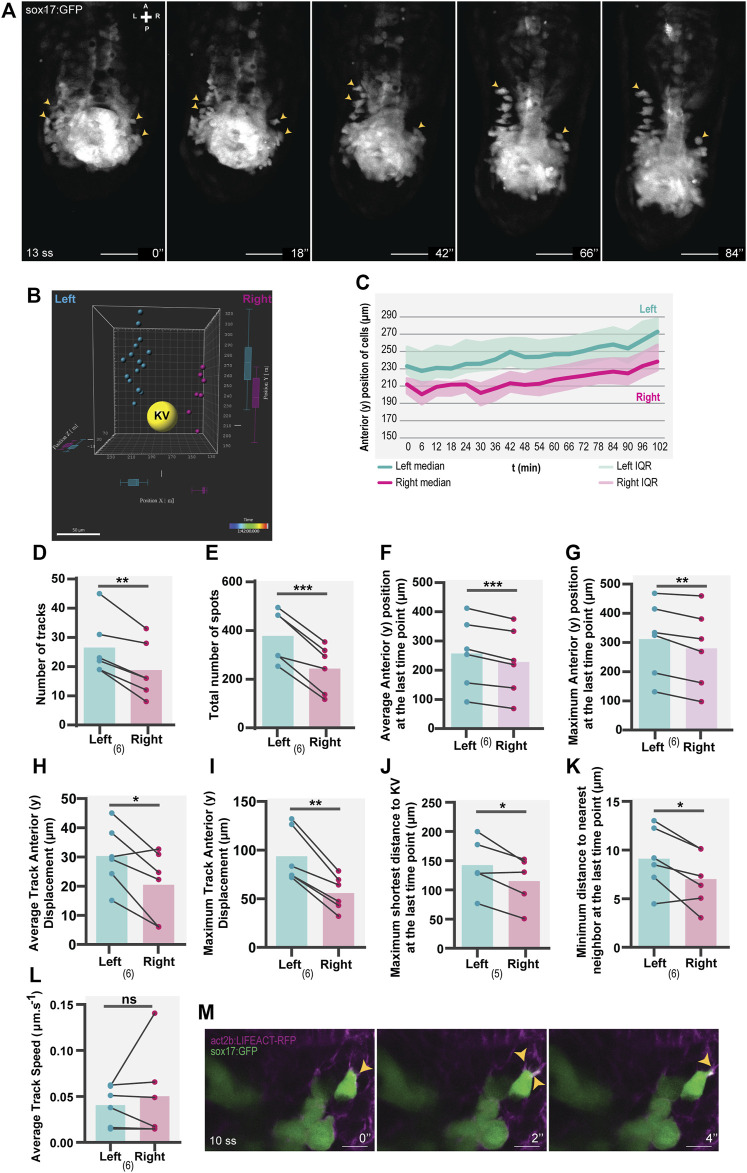
Asymmetric patterns of NKSTCs in WT embryos. **(A)** Time-lapse images of selected time points of a *Tg(sox17:GFP)* embryo starting at 13 ss and ending at 18 ss. Yellow arrowheads indicate the more anterior positions on the left and right sides. The elapsed time is indicated in minutes (‘) at the bottom-right. Scale bar = 50 µm. **(B)** 3D distribution of manually tracked cells at the last time-point imaged. Left spots are shown in cyan, right in magenta, and the KV lumen in yellow. **(C)** Dynamics of the anterior positions at consecutive time points on the left (cyan) and right (magenta) sides. The thick lines represent the medians. **(D–L)** Left vs right parameters extracted from cell tracking data, cyan represents the left side, and magenta represents the right side. *t*-test paired comparisons and IQR means interquartile range. Bars represent mean values and dots represent individual embryos. *corresponds to a *p*-value <0.05; ** to a *p*-value <0.005 and *** to a *p*-value <0.0005. **(M)** Lamellipodium-like (yellow arrowheads) structures in NKSTCs observed in *Tg(act2b:LIFEACT-RFP);Tg(sox17:GFP).* Scale bar = 10 µm. Axes indicate A, anterior; P, posterior; L, left; R, right. N is shown in parentheses.

Using the Tg(sox17:GFP) line (first described by Sakaguchi et al. (2006)) as a KV reporter, we observed a distinct group of sox17:GFP^+^ cells in the vicinity of the KV, which presented the following properties: i) asymmetric pattern of anterior migration along the midline axis (Figure 1A); ii) cell protrusions, denoting potential anterior migratory behavior; and iii) shape, size, and position distinct from that of the gut precursors. The most striking feature of these migratory cells is that they reach more anterior positions on the left side. Thus, we investigated whether sox17:GFP^+^ cells are affected by the laterality pathway. First, to identify the tracked cells, we compared their expression pattern of *sox17* mRNA by whole mount *in situ* hybridization with the sox17:GFP^+^ cells from bud stage to 14 ss. We concluded that *sox17* mRNA is strongly expressed in DFCs at the bud stage. However, we did not find any cells expressing *sox17* mRNA in the KV at 8 ss or close to the KV at 8 or 13-14 ss (Supplementary Figure S1). Therefore, the cells of interest were likely to be labeled by *sox17* promoter leakage (outside the KV), GFP perdurance (such as in the KV cells), or both. Nevertheless, this transgenic line inadvertently highlights a group of cells close to and in the same focal plane as KV, displaying an intriguing asymmetric pattern along the midline axis. Therefore, we decided to continue our study by using the *sox17:GFP* transgenic line as a tailbud cell reporter. For simplicity, we named the cells of interest: Non-KV Sox17:GFP^+^ Tailbud Cells or NKSTCs.

### Live imaging of NKSTCs revealed asymmetric patterns regarding the LR axis

We observed that NKSTCs exhibited migratory behavior towards the anterior side, with an asymmetric trend towards the left side (Figure 1A) (Supplementary Video S1). By 3D tracking these cells over time (Supplementary Video S2), we found an asymmetry in the number of tracks on each side of the KV (Figure 1D; *p*-value = 0,0024) and the total number of cells on the left side (Figure 1E; *p*-value = 0,0006). Thus, we concluded that the first asymmetric pattern was the presence of more cells on the left side, moving towards the anterior of the embryo. Over time, the cells on the left side acquired more anterior positions than those on the right (Figure 1B, C), as shown by their larger average and maximum anterior (y) positions (Figure 1F, *p*-value = 0,0006; Figure 1G; *p*-value = 0,0045) and the increased average and maximum track anterior (y) displacement (Figure 1H, *p*-value = 0,0215; Figure 1I, *p*-value = 0,0016), at the final time point. As a reference point, we analyzed the distance of the cells to the center of the KV and found that, at the final time point, the cells on the left side showed a higher maximum shortest distance to the KV than those on the right (Figure 1J; *p*-value = 0.0360). This observation meant that in each embryo analyzed in the 3D space, there were cells further distanced from the KV on the left side than on the right side. We also measured the minimum distance to the nearest neighbor at the last time point imaged, and again, this distance was larger on the left side than on the right (Figure 1K; *p*-value = 0.0263).

To assess whether NKSTCs were actively migrating, we used the *Tg(sox17:GFP);Tg(act2b:LIFEACT-RFP)* double transgenic and observed F-actin dynamics, the presence of lamellipodia and filipodia characteristics of migratory cells (Figure 1L). We then evaluated the migration speed of NKSTCs and found no differences in the average track speed (Figure 1M; *p*-value = 0.5304). Consequently, we concluded that different rates of motion were not the cause for the left-sided cells to reach more anterior positions.

**FIGURE 2:**
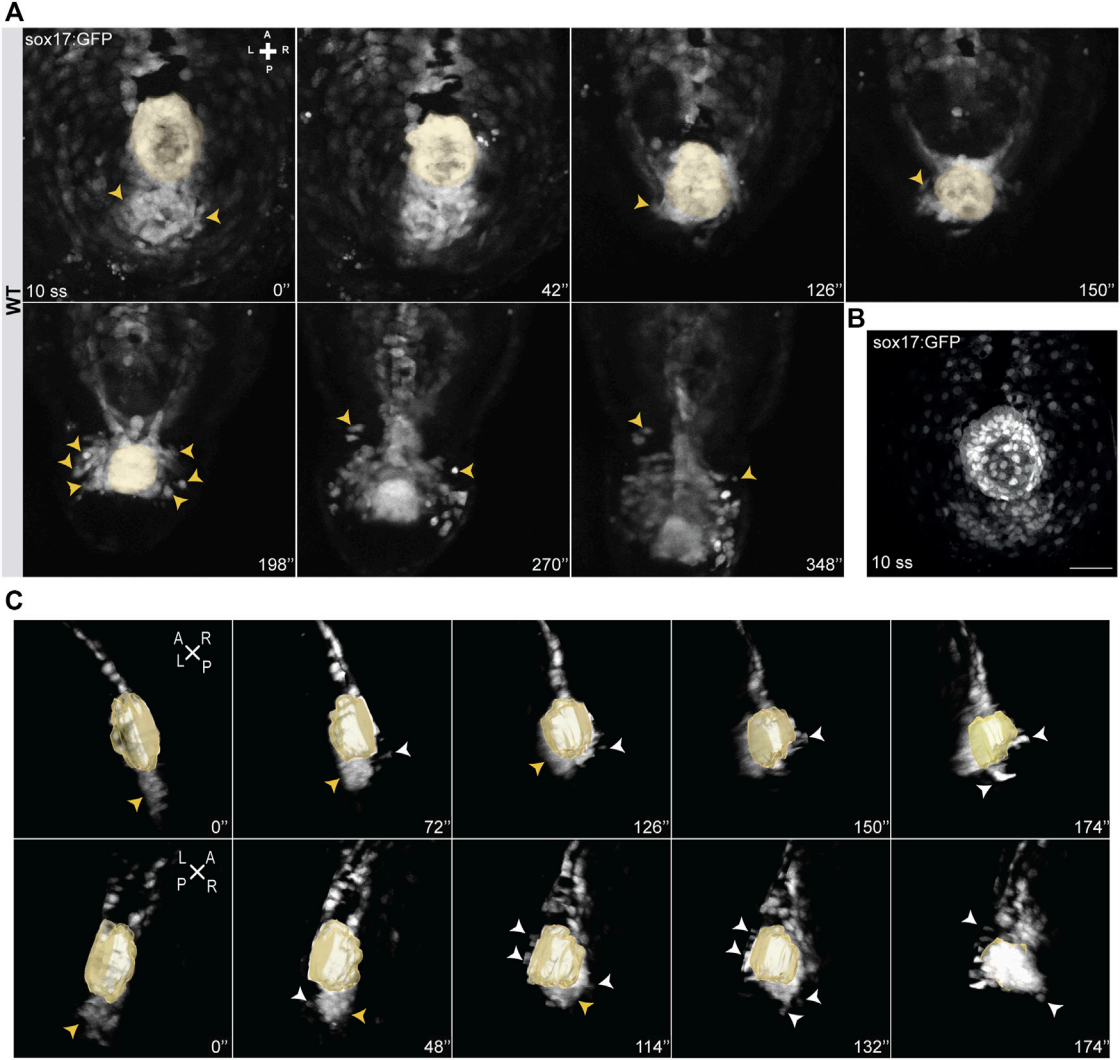
Snapshots from time-lapse live imaging of NKSTCs. **(A)** Time-lapse imaging of a WT Tg (sox17:GFP) embryo, starting at 10 ss, showing NKSTCs movement from a posterior cluster aligned with the KV. NKSTCs are firstly indicated by yellow arrowheads in the cluster. In the following snapshots, the yellow arrowheads show the first cells that exit the cluster and migrate towards the anterior side from either the left or right side. **(B)** Maximum intensity projection of a 10 ss *Tg(sox17:GFP)* fixed embryo showing the ´posterior cluster’ aligned with the KV, scale bar = 50 µm. **(C)** 3D lateral views of the KV and posterior cluster at 10 ss. Left, yellow arrowheads; right, white arrowheads. Elapsed time is indicated in minutes (‘) at the right and axes are indicated: A, anterior; P, posterior; L, left; R, right. N is shown in parentheses.

Next, we imaged NKSTCs at earlier developmental stages to determine whether we could track the establishment of the asymmetric pattern mentioned above. At 9-10 ss we observed a cluster of NKSTCs localized posterior to the KV (Figure 2A) (Supplementary Video S3) and confirmed this at 10 ss in fixed embryos for better image resolution (Figure 2B). Cell morphology in the cluster was different from that of the larger and highly dispersed endodermal cells, which later will form the gut tube. We determined that the aggregate we named the “posterior cluster” comprised an average of 42 NKSTCs (x ® = 42,33; *σ* = 8,41; *N* = 9). We observed that during the extension of the embryo, the KV is aligned with the “posterior cluster” (Supplementary Video S4), and as it approaches it, causes cells from the cluster to start leaving it in an alternating manner, either from the left or right side towards the anterior of the embryo. Again, we observed that NKSTCs acquired more anterior positions on the left side (Figures 2A–C), matching our first observation (Figure 1A). Therefore, the KV, the zebrafish LRO, appeared to be the driving force for the NKSTCs’ departure from the “posterior cluster” (Supplementary Video S4). Whether the cue triggered by the KV is mechanic or molecular is yet to be tested. Next, we manipulated the LR pathway and tested whether the migratory behavior of NKSTCs was affected. In *dand5* ^*−/−*^ embryos, the asymmetric localization of NKSTCs is lost.

**FIGURE 3:**
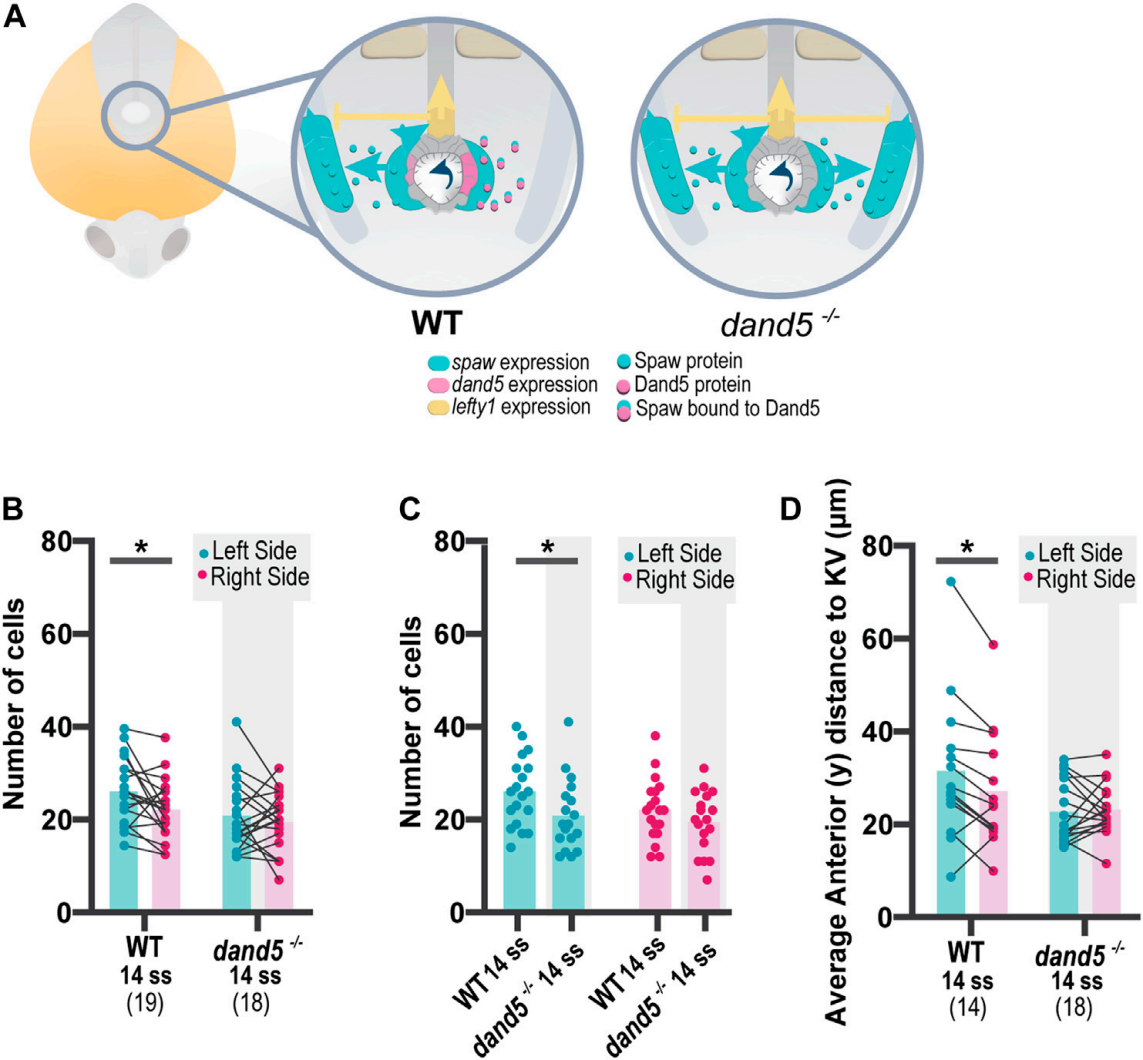
Quantification of NKSTCs distribution patterns in WT and *dand5* ^*−/−*^ embryos. **(A)** Schematic representation of early asymmetric establishment in WT and *dand5* ^*−/−*^ embryos. In the absence of *dand5* expression, Spaw freely travels to both sides of the LPM, where it activates its own expression and its inhibitor *lefty1* at the midline. **(B)** Quantification of left-right differences in the number of NKSTCs at 14 ss in WT and *dand5* ^*−/−*^ embryos. **(C)** Quantification of differences in the number of NKSTCs observed on each side of the embryos at 14 ss in WT and *dand5* ^*−/−*^ embryos. **(D)** Quantification of left-right differences observed in the anterior distance to the KV based on the 3D coordinates of NKSTCs at 14 ss in WT and *dand5* ^*−/−*^ embryos. Cyan represents the left side, and magenta represents the right side. *t*-test paired and unpaired comparisons. Bars represent mean values, and dots represent individual embryos. * Corresponds to a *p*-value <0.05.

To test how NKSTCs respond to manipulation of LR cues, we raised *dand5* ^*−/−*^ embryos on a *Tg*(*sox17:GFP*) background. In *dand5* ^*−/−*^ embryos, *dand5* mRNA expressed in the KV is absent, as is the secreted Dand5 protein (Montague et al., 2018). Therefore, in *dand5* ^*−/−*^ mutants, Southpaw could travel freely to both sides of the embryo, as shown in the diagram (Figure 3A), confirmed in our laboratory (data not shown) and by Montague et al. (2018). We predicted that if the sox17:GFP transgenic reporter line was Nodal-dependent, pronounced anterior migration of NKSTCs would be observed bilaterally.

We tested for differences between WT and *dand5* ^−/−^ embryos at 14 ss by acquiring the 3D coordinates of each cell detached from the cluster. Previous WT live imaging (Figure 1) indicated the presence of more cells on the left side; we confirmed the same pattern in WT fixed embryos at 14 ss (Figure 3B, *p*-value = 0.0262). However, for *dand5* ^*−/−*^ embryos, we observed a different scenario: at 14 ss, there was no difference in the number of cells between the left and right sides (Figures 3B,C, *p*-value = 0.4605). In addition, at 14 ss, WT embryos showed a higher average anterior distance to the KV on the left side (Figure 3D, *p*-value = 0.0162); whereas in *dand5* ^*−/−*^ mutants, there was no significant difference in the distance to the KV between the left and right sides. Furthermore, paired comparisons suggested randomization (Figure 3D) without denoting any pronounced anterior bilateral migration or right-sided increase in anterior migration, as expected in the presence of bilateral spaw expression.

**FIGURE 4:**
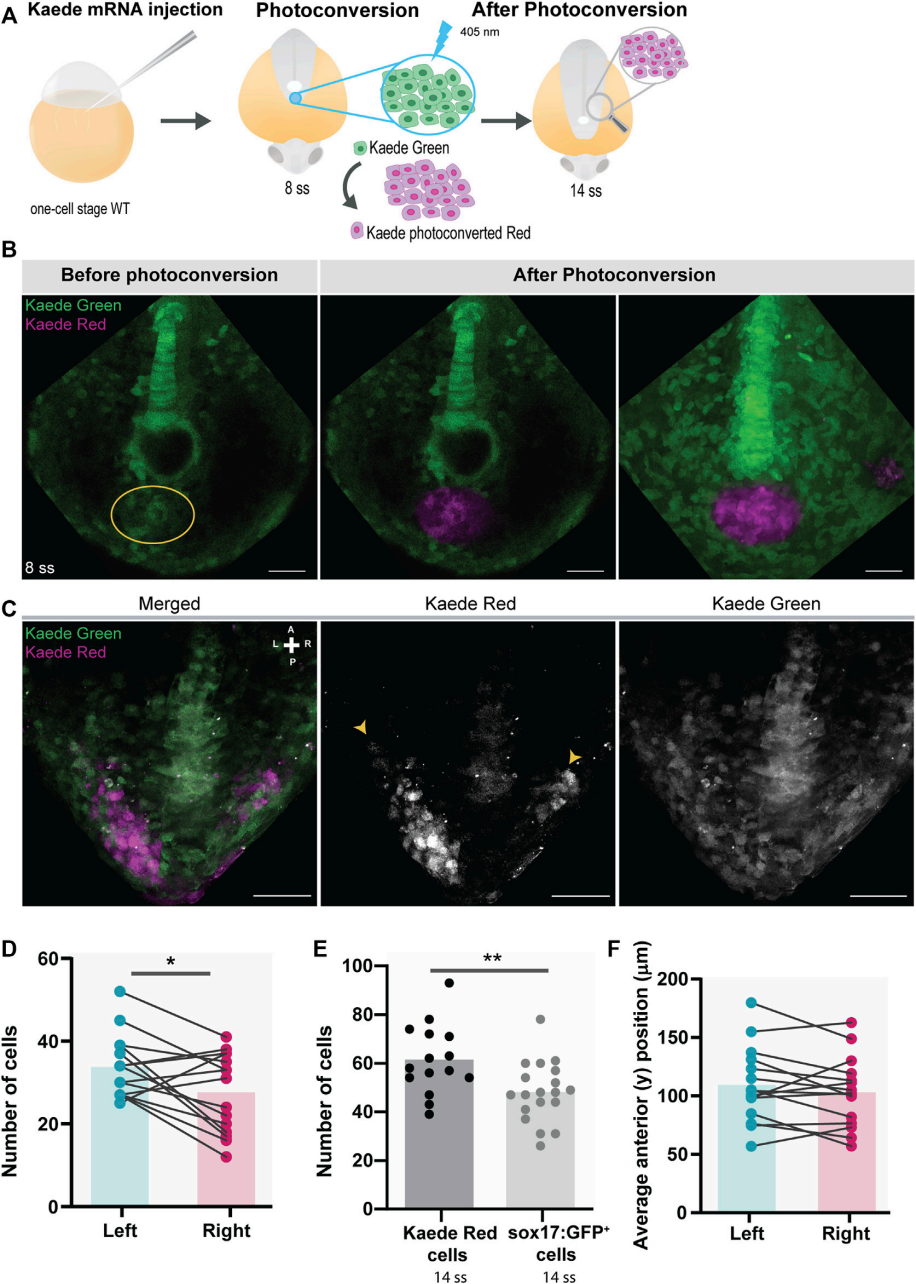
Testing for the presence of left-right asymmetries independent of the *Tg(sox17:GFP)* reporter line. **(A)** Schematic representation of the setup used to follow specific tailbud cells using the photoconvertible protein, Kaede. One-cell stage WT embryos were injected with *Kaede* mRNA. The ‘posterior cluster’ region was blindly photoconverted at 8 ss embryos using UV light. At 14 ss, embryos were screened for the location of Kaede photoconverted cells. **(B)** Before and after Kaede photoconversion of the ‘posterior cluster’ region at 8 ss in WT embryos. The KV middle focal plane was used as a reference (note that photoconversion can happen along the axial cone of illumination of the confocal, though). The maximum intensity projection shows the total area of the photoconverted cells. Kaede green cells are shown in green, and Kaede photoconverted cells are shown in magenta. Scale bar = 50 µm. **(C)** Position of the Kaede photoconverted cells in the dorsal view of a 14 ss WT embryo. Yellow arrowheads indicate the more anterior positions of the photoconverted cells on both the left and right sides. Scale bar = 100 µm. **(D)** Paired comparison of the number of photoconverted cells on the left and right side at 14 ss. **(E)** Quantification of the differences in the total number of photoconverted cells and NKSTCs at 14 ss. Dark gray represents photoconverted cells and light gray represents NKSTCs. **(F)** Paired comparison of the average anterior positions of photoconverted cells on the left and right sides of 14 ss. Cyan represents the left side and magenta represents the right side. Bars represent mean values and dots individual embryos. * corresponds to a *p*-value <0.05 and ** to a *p*-value <0.005.

To further validate the use of the *Tg(sox17:GFP)* line, we tested for asymmetries in tailbud cell movement using a method independent of the reporter line. We repeated the experiments by photoconverting the region of the “posterior cluster” at 8 ss in *Kaede*-injected embryos and assessed the positions of the photoconverted cells on the left and right sides at 14 ss, as shown in the diagram (Figure 4A). Again, we observed a more significant number of cells on the left side (*p*-value = 0.0266) (Figures 4B–D). Nevertheless, the number of Kaede photoconverted cells found in the tails of 14 ss embryos was higher than that of sox17:GFP^+^ cells (NKSTCs) (*p*-value = 0.0068) (Figure 4E). We cannot discard the possibility that there are photoconverted cells above and below the proximity of the KV (i.e., along the *z*-axis), since we used a conventional confocal microscopy system to perform the photoconversion which effectively creates an axial cone of illumination, and not a confined volume (Figure 4B). In Kaede photoconverted embryos, we observed that 9 embryos out of 15 (60%) reached more anterior cell positions on the left side (Figure 4F; *p*-value = 0.2148). Therefore, Kaede photoconversions validated the higher numbers of cells on the left side observed by the sox17:GFP reporter but failed to provide statistical significance to the left anterior bias.

### *dand5* ^*−/−*^ mutant cells migrate bilaterally synchronized from the posterior cluster

**FIGURE 5:**
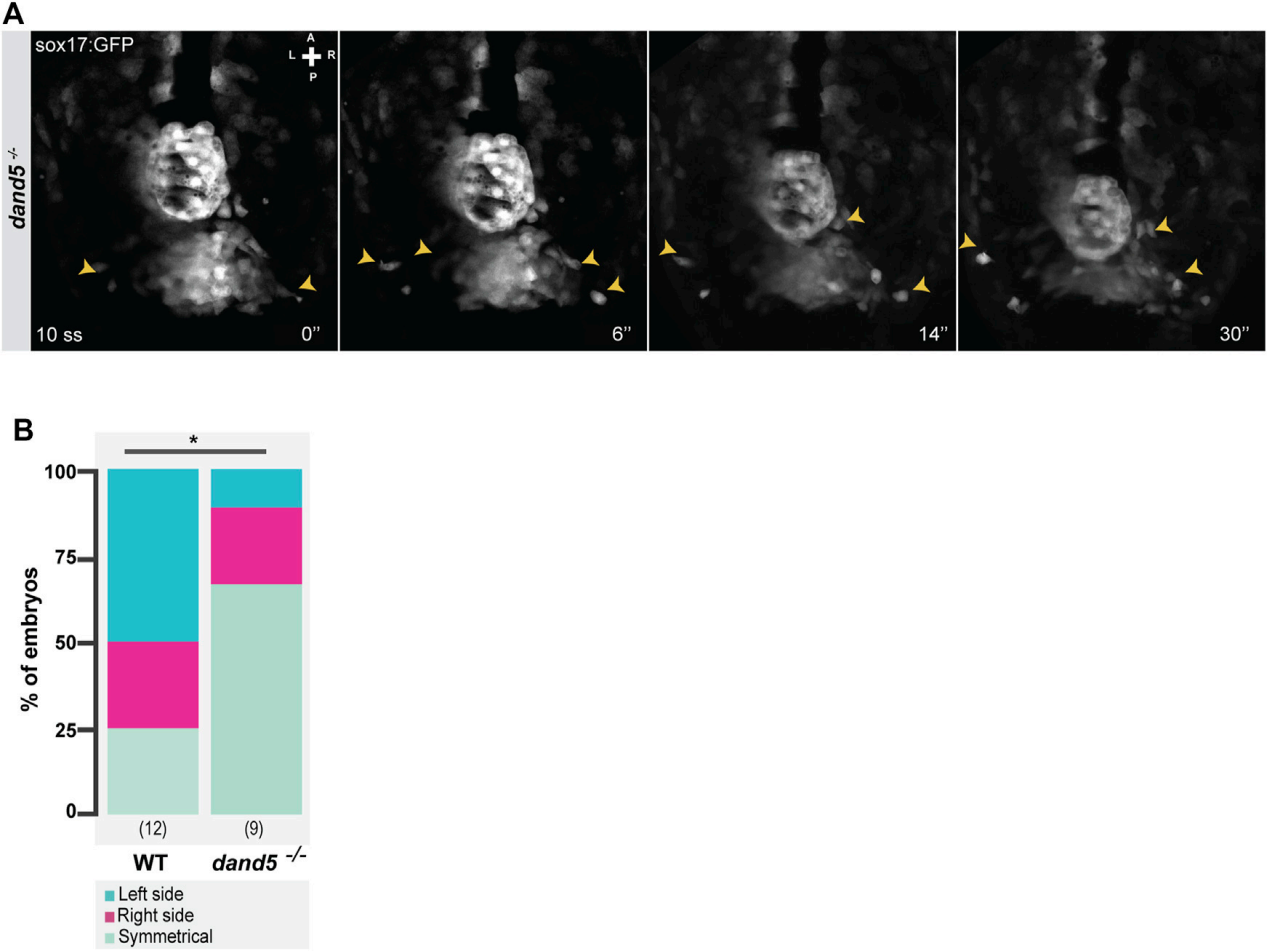
Snapshots from time-lapse live imaging of NKSTCs leaving the posterior cluster. **(A)** Time-lapse imaging of a *dand5* ^*−/−*^ embryo raised on a *Tg*(*sox17:GFP*) background, starting at 10 ss. **(B)** Quantification of the first side from which NKSTCs departed from the posterior cluster in WT and *dand5* ^*−/−*^ embryos. Chi-square test. * corresponds to a *p*-value <0.05. Elapsed time is indicated in minutes (‘) at the bottom right, and the axes are indicated: A, anterior; P, posterior; L, left; R, right. N is shown in parentheses.

Next, having established that the NKSTCs come from the “posterior cluster”, we quantified if the asymmetry seen in their destination at 14 ss was first visualized by an asymmetric departure from the “posterior cluster”. We found that in 50% of the analyzed WT embryos (*N* = 12), the first cells to depart from the posterior cluster were from the left side, 25% from the right side, and 25% from both sides simultaneously. However, in *dand5* ^*−/−*^ embryos (Figure 5A), 67% of NKSTCs departed simultaneously from both sides, 22% left first from the right side, and 11% from the left side (Supplementary Video S5; *N* = 9; Figure 5B). These results showed that cell behavior differed between WT and *dand5* ^*−/−*^ embryos.

As cells in the “posterior cluster” appear to respond to the KV position to exit towards the anterior side of the embryo, we tested whether the predominantly symmetrical behavior in *dand5* ^*−/−*^ embryos could be a result of changes in KV morphology. We evaluated KV lumen morphology in both 8 ss WT and *dand5* ^*−/−*^ embryos raised in a sox17:GFP background (Supplementary Figures S3A, B). Both the volume and area of the KV lumen did not differ between WT and *dand5* ^*−/−*^ embryos (Supplementary Figures S3C, D). Nevertheless, *dand5* ^*−/−*^ embryos presented more spherical KVs (Supplementary Figure S3E). Consequently, we established that symmetric cell exit in *dand5* ^*−/−*^ embryos is not triggered by differences in the KV volume.

### NKSTCs have a somitic fate

**FIGURE 6:**
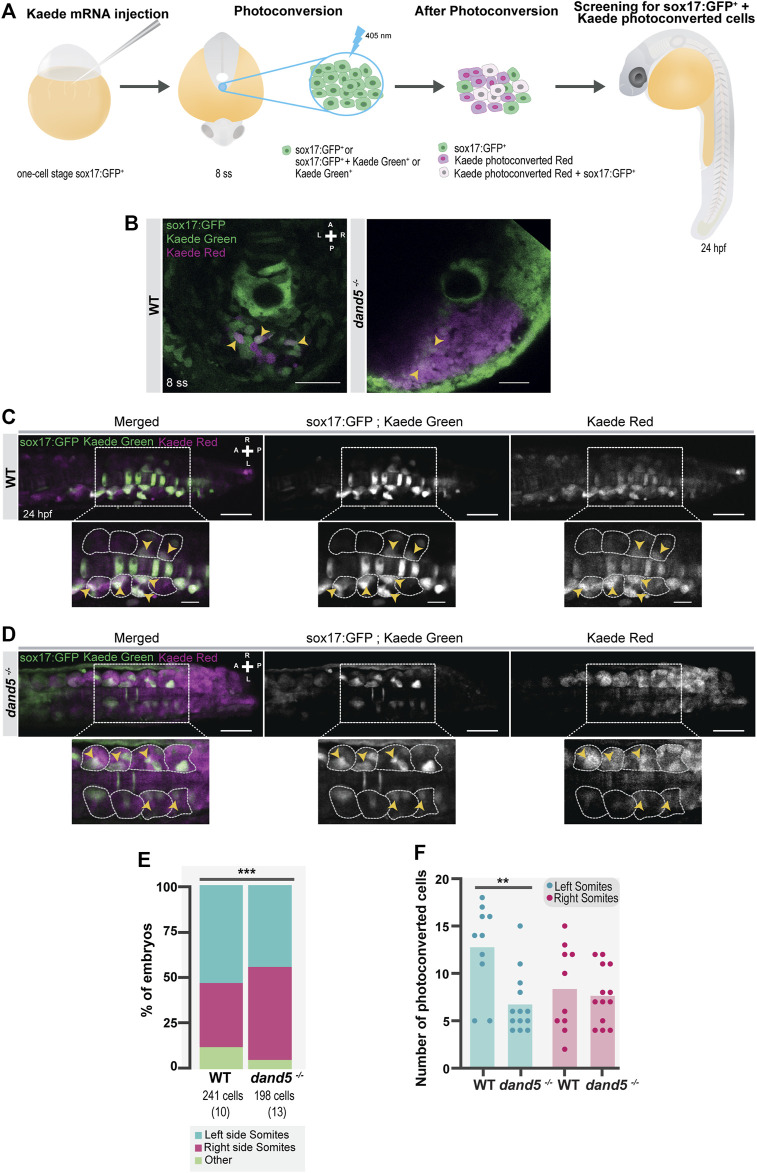
NKSTCs fate mapping. **(A)** Schematic representation of the fate map setup using the photoconvertible protein, Kaede. One-cell stage Tg (sox17:GFP) embryos were injected with *Kaede* mRNA. The “posterior cluster” was photoconverted at 8 ss embryos using UV light. After photoconversion, there were three populations of cells in the region of interest: sox17:GFP^+^ (green), Kaede photoconverted (magenta), and Kaede photoconverted colocalized with sox17:GFP^+^ (white). At 24 hpf, the embryos were screened for the location of Kaede photoconverted colocalized with sox17:GFP^+^ cells. The same procedure was used in *dand5* ^*−/−*^ embryos in *Tg*(*sox17:GFP*) background. **(B)** Kaede photoconversion of the “posterior cluster” at 8 ss in WT and *dand5* ^*−/−*^ embryos. Kaede photoconverted is in magenta; sox17:GFP^+^ and Kaede not photoconverted are in green and Kaede photoconverted colocalized with sox17:GFP^+^ cells are shown in white. Scale bar = 50 µm. **(C)** Dorsal view of a *Tg(sox17:GFP)* 24 hpf Kaede photoconverted embryo. Scale bar = 50 µm. Magnification shows a region of interest where NKSTCs (yellow arrowheads) are located at both the left and right somites (yellow arrowheads). The somites are outlined by light grey dashed lines. Scale bar = 20 µm. **(D)** Dorsal view of a *dand5* ^*−/−*^; *Tg*(*sox17:GFP*) 24 hpf Kaede photoconverted embryo. Scale bar = 50 µm. Magnification shows a region of interest where NKSTCs (yellow arrowheads) are located at both the left and right somites (yellow arrowheads). The somites are outlined by light grey dashed lines. Scale bar = 20 µm. **(E)** Quantification and comparison of the proportion of cells that incorporated somites and ‘other fates’ in both WT and *dand5* ^*−/−*^ 24 hpf embryos. Chi-square test. * corresponds to a *p*-value <0.05. **(F)** Quantification of the differences in the number of NKSTCs per somite side. Cells on the left and right sides of WT vs *dand5* ^*−/−*^. *t*-test paired and unpaired comparisons. Bars represent mean values and dots individual embryos. * corresponds to a *p*-value <0.05 and ** to a *p*-value <0.005. N is shown in parentheses.

To fate-map the NKSTCs, we photoconverted the ‘posterior cluster’ at 8 ss before NKSTCs migration (Figures 6A, B) using *Tg(sox17:GFP)* embryos in both WT and *dand5* mutants. We scored the cell fates of double-labeled sox17:GFP^+^ and Kaede photoconverted cells at 24 hpf (white cells) (Figures 6A–D). In WT embryos at 24 hpf, 88% of the photoconverted cells were found in the tail somites, and the remaining 12% were found under the category of “other fates” (*N* = 10; Ncells = 241), which included the notochord and nearby structures (Figure 6E).

In *dand5* ^*−/−*^ embryos, we found that photoconverted NKSTCs also integrated somites (94,9%; *N* = 13; Ncells = 198; Figures 6D, E). However, the proportion of acquired fates in the *dand5* ^*−/−*^ mutants differed significantly from that in the WT embryos (Figure 6E; *p*-value = 0,0009). Regarding LR cell fates within the somites, for WT embryos, 53% of photoconverted NKSTCs were found on the left and 35% on the right somites. Therefore, in a pairwise comparison, we determined that left-sided somites received more photoconverted cells (*p*-value = 0.0457). In *dand5* ^*−/−*^ mutants, 44% of NKSTCs were found on the left somites, and 50,5% were found on the right somites, which was not significantly different in a pairwise comparison (*p* = 0.4688). Moreover, in *dand5* ^*−/−*^embryos the number of cells integrating the left somites was smaller than in WT embryos (Figure 6F; *p*-value = 0.0031). Thus, we concluded that in WT embryos, NKSTCs tended to incorporate more left-sided somites than right-sided somites. In contrast, in *dand5* ^*−/−*^ embryos, this incorporation is mainly symmetric, despite a tendency towards right-sided somites.

In addition, we investigated whether NKSTCs integrate somites in an anterior asymmetric manner. To test this hypothesis, we scored the maximum anterior positions observed at 24 hpf in photoconverted NKSTCs compared with the anterior positions of Kaede photoconverted cells alone (Supplementary Figure S4A) in the same embryos. We concluded that photoconverted NKSTCs cells showed a left-sided bias (Supplementary Figure S4C) that was unattainable by scoring all Kaede photoconverted cells (Supplementary Figure S4B). As photoconversion potentially reaches more cells along the axial cone of illumination of the confocal (labeled magenta cells), it may make it more difficult to ascertain the subtle asymmetries found by observing only photoconverted NKSTCs (labeled both green and magenta).

To further understand the consequences of NKSTCs in somite formation, we examined whether there was a difference in early somite specification between WT and *dand5* ^*−/−*^ embryos. We aimed to score both *dand5* expression and *myoD*, a myogenic differentiation factor and one of the earliest markers of myogenic commitment (Weinberg et al., 1996). We performed WISH using probes for both *myoD* and *dand5*. In WT embryos, the mRNA of the two genes can be easily discerned in the tailbud because of the different 3D localization of the adaxial cells (more dorsal) and the KV (more ventral) (Supplementary Figure S5A). Moreover, in *dand5* ^*−/−*^ embryos, *dand5* mRNA is absent (Supplementary Figure S4B). Our first observation in WT embryos was that *myoD* expression in the presumptive somites was symmetrical on each side of the adaxial cells in 48% of the embryos (*N* = 27), meaning that we observed the same number of segments labeled by *myoD* on each side of the embryo.

**FIGURE 7:**
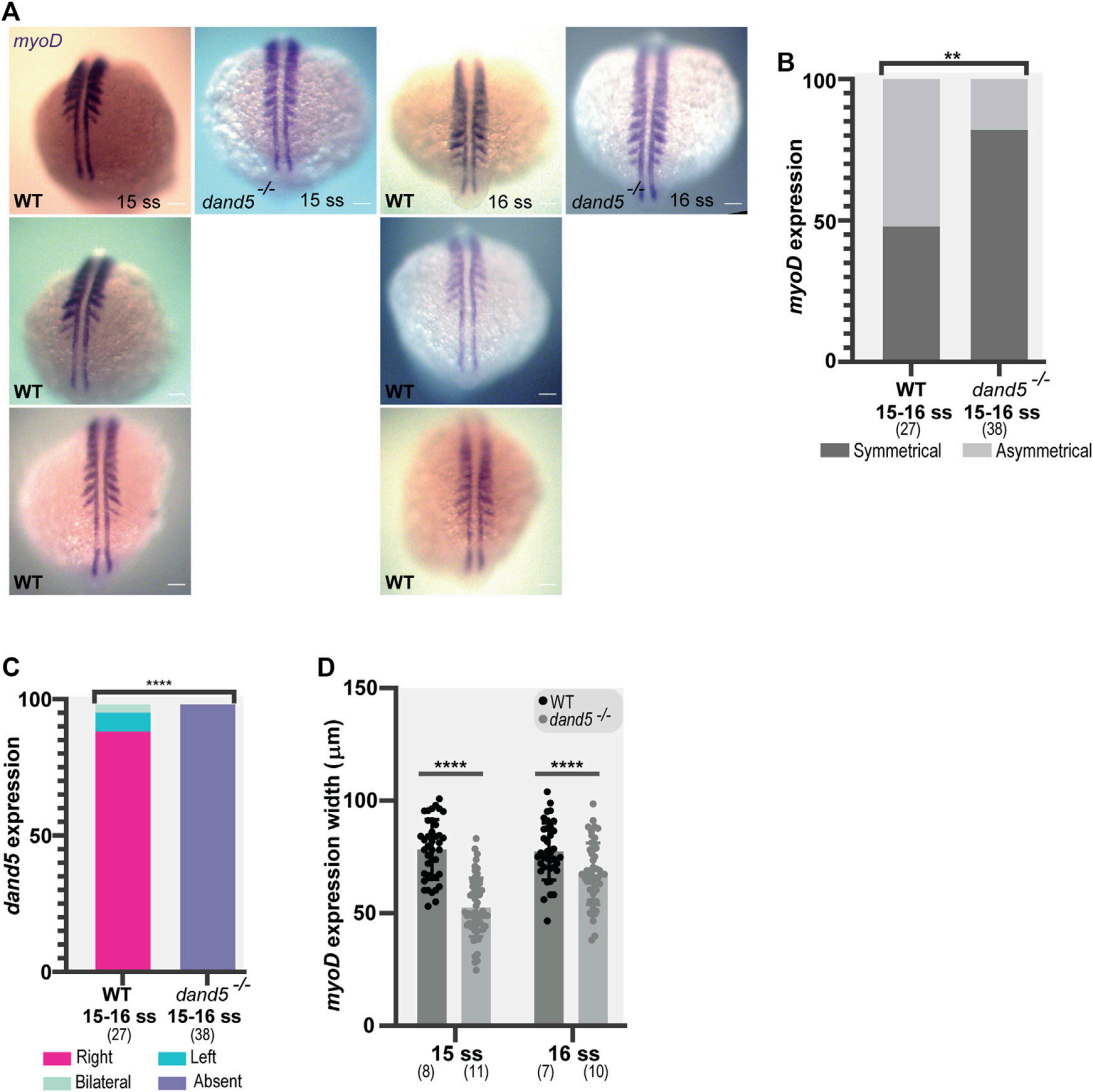
Whole-mount *in situ* hybridization for *myoD* and *dand5* expression. **(A,B)** *myoD* expression and side scoring. **(C)** Quantification of differences in *dand5* expression patterns in WT vs *dand5* homozygous mutants. The chi-squared test was used for statistical analysis. **(D)** Quantification of *myoD* width in WT and *dand5* ^*−/−*^ at 15 and 16 ss embryos. *t*-test unpaired comparison. Bars represent mean values, and dots represent individual embryos. * corresponds to a *p*-value <0 05; ** to a *p*-value <0.005; *** to a *p*-value <0.0005 and **** to a *p*-value <0.00005. Scale bar = 100 µm. The top is anterior, and the bottom is posterior. N is shown in parentheses.

However, 52% of the WT embryos showed asymmetrical *myoD* expression (Figures 7A, B). Next, we scored *dand5* expression in combination with *myoD* expression. In most *dand5* ^*−/−*^ embryos we observed a symmetrical *myoD* expression (82.8%), significantly different from the WT expression pattern (Figure 7B; *p*-value = 0.0064). We also observed that 12% of *dand5* ^*−/−*^ embryos showed fused somites at the midline at 15-16 ss (Supplementary Figures S5D, E), revealing a midline specification defect in a small portion of embryos. We then scored *dand5* expression at 15-16 ss and, as expected, found that in WT embryos, the expression was right-sided (85.19%, *N* = 27; Figure 7C). This experiment suggests that the absence of *dand5* correlates with symmetric *myoD* expression.

In addition, we observed that in *dand5* ^*−/−*^ embryos, the width of the segments labeled with *myoD* was smaller than that in WT embryos at 15 ss (*p*-value <0.0001) and 16 ss (Figure 7D; *p*-value = 0.0010). Therefore, in the absence of Dand5, the early somite specification is affected which may result in smaller somites.

### The absence of Dand5 function affects body size but not somite symmetry

**FIGURE 8:**
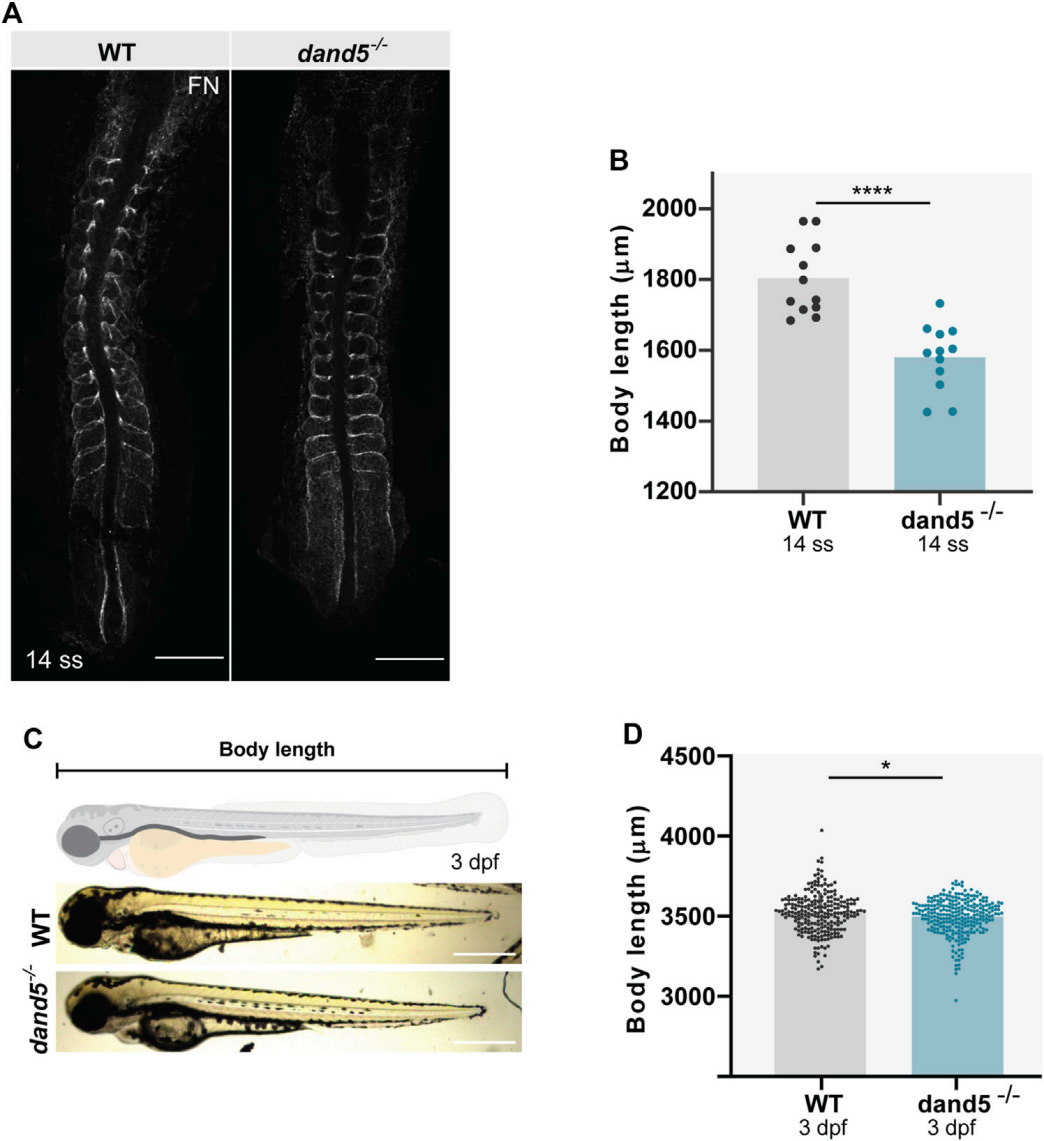
Body size and somite morphology in WT and *dand5* ^*−/−*^ embryos. **(A)** Fibronectin (FN) immunostaining shows somite borders in WT and *dand5* ^*−/−*^ embryos at 14 ss. Scale bar = 100 µm. The top is anterior; the bottom is posterior. **(B)** Comparison of WT and *dand5* ^*−/−*^ body length at 14 ss. **(C)** WT and *dand5* ^*−/−*^ 3 dpf larvae and schematic representation of body length measurements. Scale bar = 500 µm. **(D)** Comparison of body length measurements between WT (*N* = 234) and *dand5* ^*−/−*^ (*N* = 271) 3 dpf larvae. Gray represents WT embryos, and blue represents *dand5* ^*−/−*^ embryos. *t*-test unpaired comparison. Bars represent mean values, and dots represent individual embryos. * corresponds to a *p*-value <0.05; **** corresponds to a *p*-value <0.00005.

Finally, to analyze the effect of the absence of Dand5 on somite morphology, we measured somite size in *dand5* ^*−/−*^ embryos using a different approach. We performed immunostaining for fibronectin (FN), which labels somite borders at 14 ss (Figure 8A). The results showed that the body length was shorter in *dand5* ^*−/−*^ mutants (Figure 8B; *p*-value <0.0001). The somite dimensions were measured and normalized to body length (Supplementary Figures S6A–C). We established that Dand5 is essential for regular somite size and body length at 14 ss. We also tested the differences between left and right somite sizes. We found a randomized distribution of left vs right differences in both WT (Supplementary Figures S7A–C) and *dand5* ^*−/−*^ embryos (Supplementary Figures S7D–F). Therefore, we concluded that somite symmetry was not altered in either WT or *dand5* ^*−/−*^ embryos. To test whether the smaller body length found in *dand5* ^*−/−*^ embryos could affect later zebrafish size, we compared the body length of WT and *dand5* ^*−/−*^ in 3 dpf larvae. We observed that 3 dpf *dand5* ^*−/−*^ larvae were slightly smaller than WT (Figures 8C,D; *p*-value = 0,0220), however, the difference at 3 dpf was less pronounced than at 14 ss, indicating that the first observed difference in body size is likely transient. Thus, we concluded that body size might be rectified later, probably with no effect on adult fish size.

## Discussion

### *In vivo* LR asymmetries in somite precursors

In this study, we describe a population of tailbud cells (NKSTCs) that shows LR asymmetric features in cell number and migration patterns. We showed that most NKSTCs become somites with a left-sided bias. Furthermore, we established that these cells respond to LR cues by losing their migratory asymmetries in the absence of Dand5.

As previously reviewed, symmetric structures must be shielded from asymmetric cues to keep the segmentation clock and wavefront bilaterally synchronous, with two opposing gradients acting at the wavefront (Brend and Holley, 2009; Grimes, 2019). In zebrafish, *raldh2* (aldehyde dehydrogenase 1, A2) morphants and *nls* mutants (lacking RALDH2 activity) showed that a lack of RA signaling disrupted the synchronicity of somite formation by increasing the number of latest formed somites on the left side between the 6th and 13th somites (Kawakami et al., 2005). The timing of asymmetric somite formation in the absence of RA signaling is consistent with the time at which LR cues are transmitted to the LPM (Long et al., 2003). Kawakami *et al.* indicated that the asymmetric phenotype was due to the desynchronization of Notch activity on the left side, where cyclic genes such as *deltaC*, *her1,* and *her7* were out of phase between the left and right sides and showed an extension of *fgf8* expression on the right side (Kawakami et al., 2005). In the present study, we showed that 52% of WT embryos exhibited asymmetric *myoD* expression in the last-formed somites. Based on our findings, we suspect that these asymmetries are common and may result from early events that are resolved during development and have no impact on final somite differentiation. Using live imaging, we demonstrated that NKSTCs move asymmetrically from the posterior side of the embryo towards the anterior side and are biased to the left side in 50% of the embryos. We also confirmed by Kaede photoconversion and fate mapping that these cells are somite precursors that eventually incorporate somites in a left-sided biased manner although somites later become symmetrical structures. In addition, using fixed embryos, we showed that cell asymmetries in somite incorporation are due to Dand5 function. In mutants homozygous for *dand5*, NKSTCs do not assume asymmetric positions, as mentioned earlier, and incorporate somites in a randomized manner.

### A new function for the LRO

In addition to being the LR organizer, we suggest that the mechanical properties of KV could aid the morphogenetic movements of the zebrafish tailbud. The KV is a spheroid-like transient structure. Many studies have focused on the mechanical properties of zebrafish tail elongation in addition to the well-accepted role of morphogen gradients (Das et al., 2019). It has been shown that behind these morphogenetic changes are events sustained by a fluid-solid transition, where the mesodermal progenitor zone, at the posteriormost region of the tailbud, shows fluid-like tissue behavior and the PSM behaves as a solid (Mongera et al., 2018; Banavar et al., 2021). Moreover, Sanematsu et al. (2021) studied the pressure and shear stress exerted by tailbud cells on KV reshaping. In close similarity to what we show here, the posterior cluster appeared to respond to the proximity of KV. We presume that the KV may be pushing these cells, leading them to detach and begin their anterior migratory behavior. Further studies are required to test whether KV also offers mechanical cues for the anterior migration of tailbud cells.

### Dand5 is involved in *myoD* expression patterns

*Dand5* and *myoD* expression overlap during embryonic segmentation. *dand5* is reported to be expressed throughout the segmentation period from 1–25 ss (White et al., 2017) and expression peaks are higher between 14–19 ss (White et al., 2017) when asymmetry is already established at the LPM (Long et al., 2003). Moreover, its expression is limited to KV-derived cells (Ikeda et al., 2022). These findings suggest that Dand5 secreted by KV cells, besides breaking embryonic symmetry may play an important role in a yet unknown process. On the other hand, *myoD* expression is first detected from 75% epiboly closer to the organizer and starts in the adaxial cells on each side of the future notochord before somite formation. Immediately after somite formation, *myoD* is expressed bilaterally in the posteriormost region of the somites. In this study, we noticed that in WT embryos, *myoD* expression fluctuates as it is not perfectly aligned bilaterally 50% of the times, one side of the embryo showing one more presumptive somite marked by *myoD* expression. In contrast, *dand5* ^*−/−*^ embryos always displayed symmetric *myoD* expression. Therefore, the absence of Dand5 may prevent fluctuations in LR during myogenic differentiation. *myoD* is a myogenic regulator factor (MRFs), along with *myf5* and *mrf4* (Pownall et al., 2002), and its expression represents a commitment to myogenic fate (Devoto et al., 1996). Here, we showed that there is noise in the process of myogenesis in the sense that the left and right sides are not always aligned. Dand5 secreted protein, encoded by the first known asymmetric gene, seems to be responsible for generating some asymmetry in presumptive somites from the 13 to 15 somite stage. To understand the extent to which Dand5 interacts with myogenic differentiation and somite specification, further evaluation of the expression patterns of other myogenic differentiation factors and clock genes in the absence of *dand5* expression is required.

### Embryos are smaller in the absence of Dand5

The flow of cells through the PSM has been proposed to control somite size (Fior et al., 2012). Both *tbx16* and *msgn1* are responsible for the differentiation and movement of mesoderm progenitor cells from the tailbud to the PSM (Fior et al., 2012; Yabe and Takada, 2012; Manning and Kimelman, 2015). In the absence of *msgn1,* somites are consequently smaller due to decreased cell flow (Fior et al., 2012) and *tbx16* and *msgn1* mutants show a deficient exit from the mesodermal progenitor zone due to unproductive lamellipodia and loss of directed movement to the anterior side (Manning and Kimelman, 2015). Such publications support our results because, despite being transient phenotypes, *dand5* ^−/−^ embryos showed smaller somites, proportional to their shorter body length.

Moreover, we observed fewer cells exiting from the ‘posterior cluster’ in the *dand5* mutants and fewer photoconverted cells were found in somites at 24 hpf.

The tailbud is an overly complex region of diverse molecular interactions: Wnt and FGF signaling cooperate to induce mesodermal commitment from the neuromesodermal population, thereby positively regulating *tbx16* and *msgn1* (Goto et al., 2017). Controlled levels of BMP signaling in the zebrafish tail are also vital for the correct exit of cells from the tailbud and differentiation into tail somites, high levels of BMP inhibiting cell exit from the tailbud (O’Neill and Thorpe, 2013). Nodal is also essential for tailbud cell exit, since *oep*; *tbx16* mutants lack somites (Griffin and Kimelman, 2002), but not by inhibiting BMP signaling (O’Neill and Thorpe, 2013). In zebrafish, Dand5 was shown to inhibit all three Nodals: *sqnt*, *cyc*, and *spaw*, and to exert weak BMP inhibition given the absence of a dorsalized phenotype when overexpressed (Hashimoto et al., 2004). We hypothesized that Dand5 could regulate tailbud cell exit to the PSM through its inhibitory action on either Nodal or BMP signaling. Dand5 could inhibit BMP locally in cells close to the KV and KV-derived cells, where *dand5* expression persists; therefore, Dand5 could maintain optimal levels of BMP signaling and cooperate with *chordin* and *noggin* (Dal-Pra et al., 2006; Von Der Hardt et al., 2007) controlling cell exit from the tailbud.

Alternatively, in *dand5* ^*−/−*^ embryos, earlier *spaw* expression, seen in KV adjacent cells (Montague et al., 2018), could affect the induction of somite precursors to leave the tailbud, accelerating the initial cell flow into the PSM and consequently reducing the number of cells in the tailbud niche. These interpretations, formulated as two hypotheses, highlight the potential role of Dand5 in somite precursor specification in addition to LR establishment. Thus, to verify how tailbud cell exit is affected, we highlight the need for further studies regarding BMP and Wnt signaling, in addition to assessing *msgn1* and *tbx16* expression in the zebrafish tailbud under the manipulation of *dand5* expression.

Our report on the link between the first asymmetric gene in LR establishment, *dand5*, in zebrafish tailbud movements sets the foundation for studying the involvement of other genes of the LR cascade in this context. Since, *spaw* is asymmetrically expressed in the LPM due to inhibition by Dand5, further studies on tailbud cell movement, myogenic differentiation, and tail size in *spaw* ^*−/−*^ and *dand5* ^*−/−*^;*spaw* ^*−/−*^ (Montague et al., 2018) embryos would aid in evaluating the link between LR patterning and tailbud events.

**FIGURE 9:**
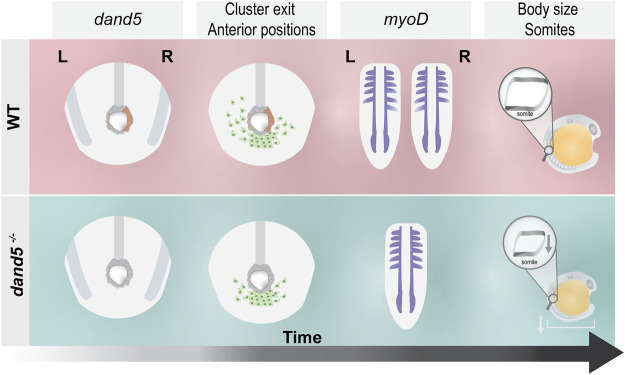
Schematic illustration of the main findings of this study. In WT embryos, *dand5* is expressed on the right side of the LRO, NKSTCs exit the posterior cluster first from the left side in 50% of the embryos, and *myoD* is symmetrical in the same proportion of embryos. In *dand5* ^*−/−*^ mutants, which lack *dand5* expression, NKSTCs simultaneously depart from the posterior cluster from the left and right sides and acquire symmetrical positions. Later, *myoD* presumptive somite expression becomes symmetric, accordingly. In addition, in *dand5* mutants, the width of the *myoD* segments was significantly shorter and the body length was decreased.

As a final illustrative summary, we describe the different steps affected by the absence of Dand5 without inferring a causal relationship (Figure 9).

## Data Availability

The raw data supporting the conclusion of this article will be made available by the authors, without undue reservation.
