# Supplementary figures and images for: Dand5 is involved in zebrafish tailbud cell movement
Source: Front Cell Dev Biol. 2023 Jan 9;10:989615. doi: 10.3389/fcell.2022.989615 (PMC9869157; doi:10.3389/fcell.2022.989615)

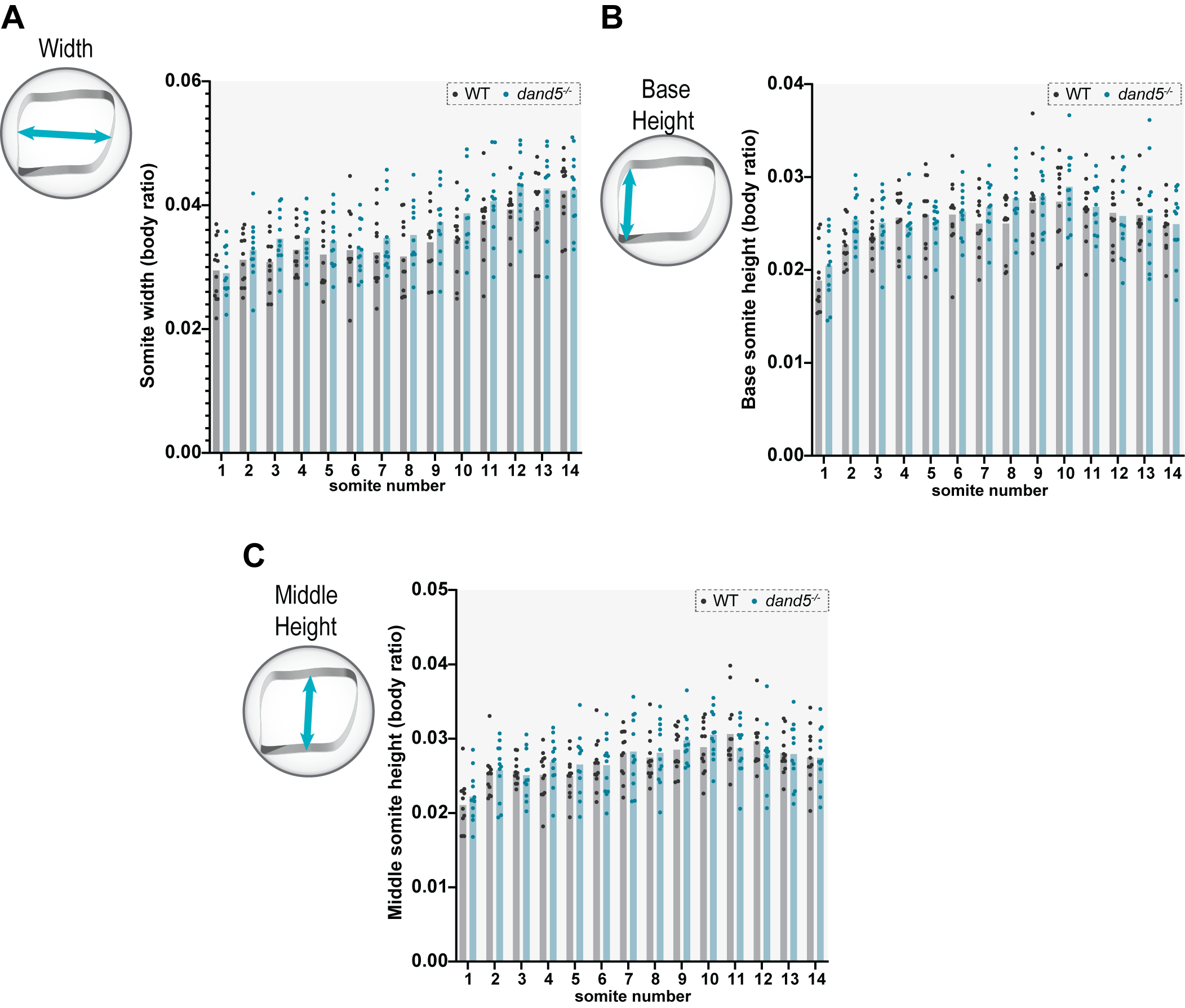

Supplement: Supplementary file 3 [file Image6.TIF]

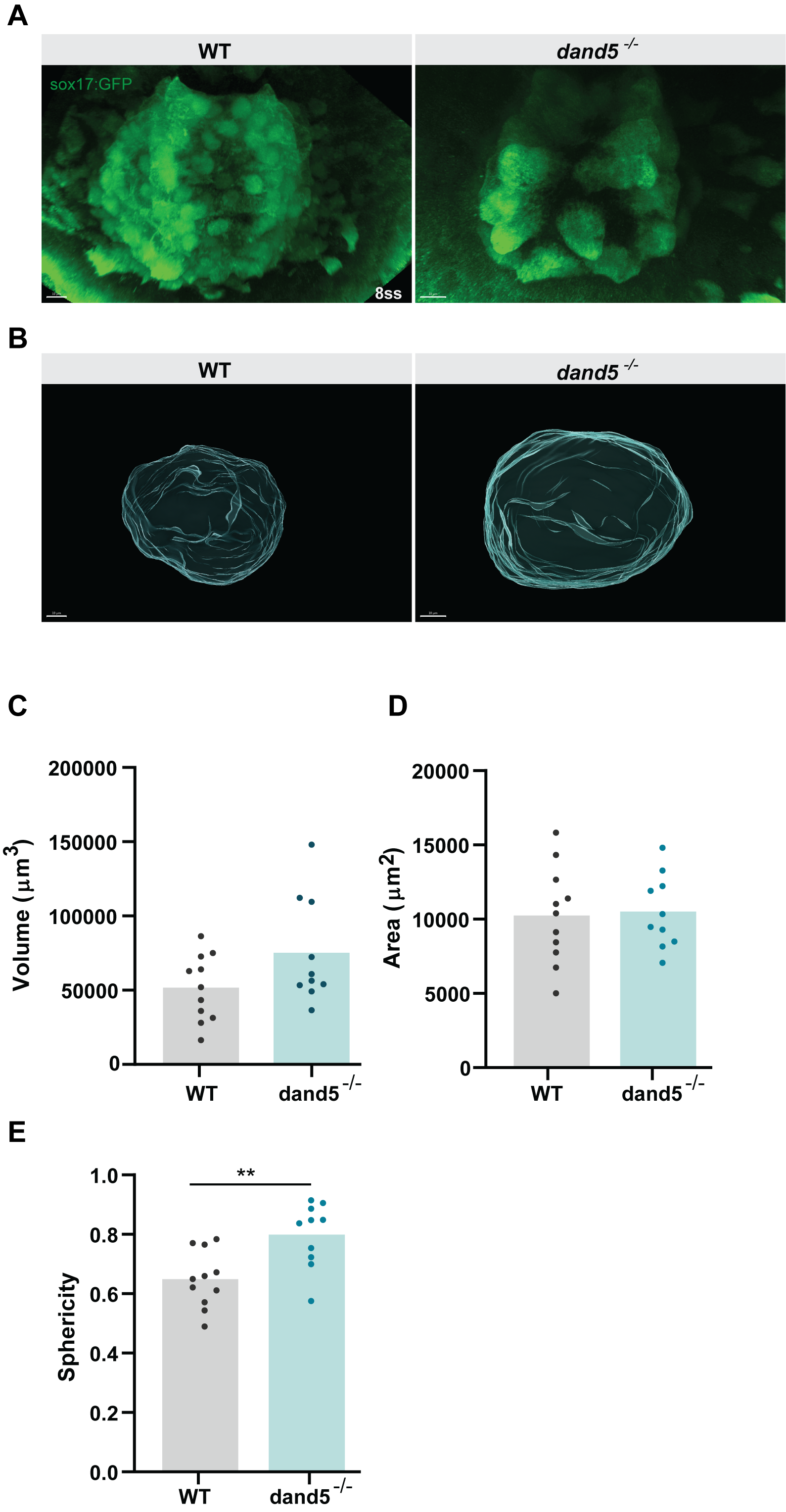

Supplement: Supplementary file 4 [file Image3.TIF]

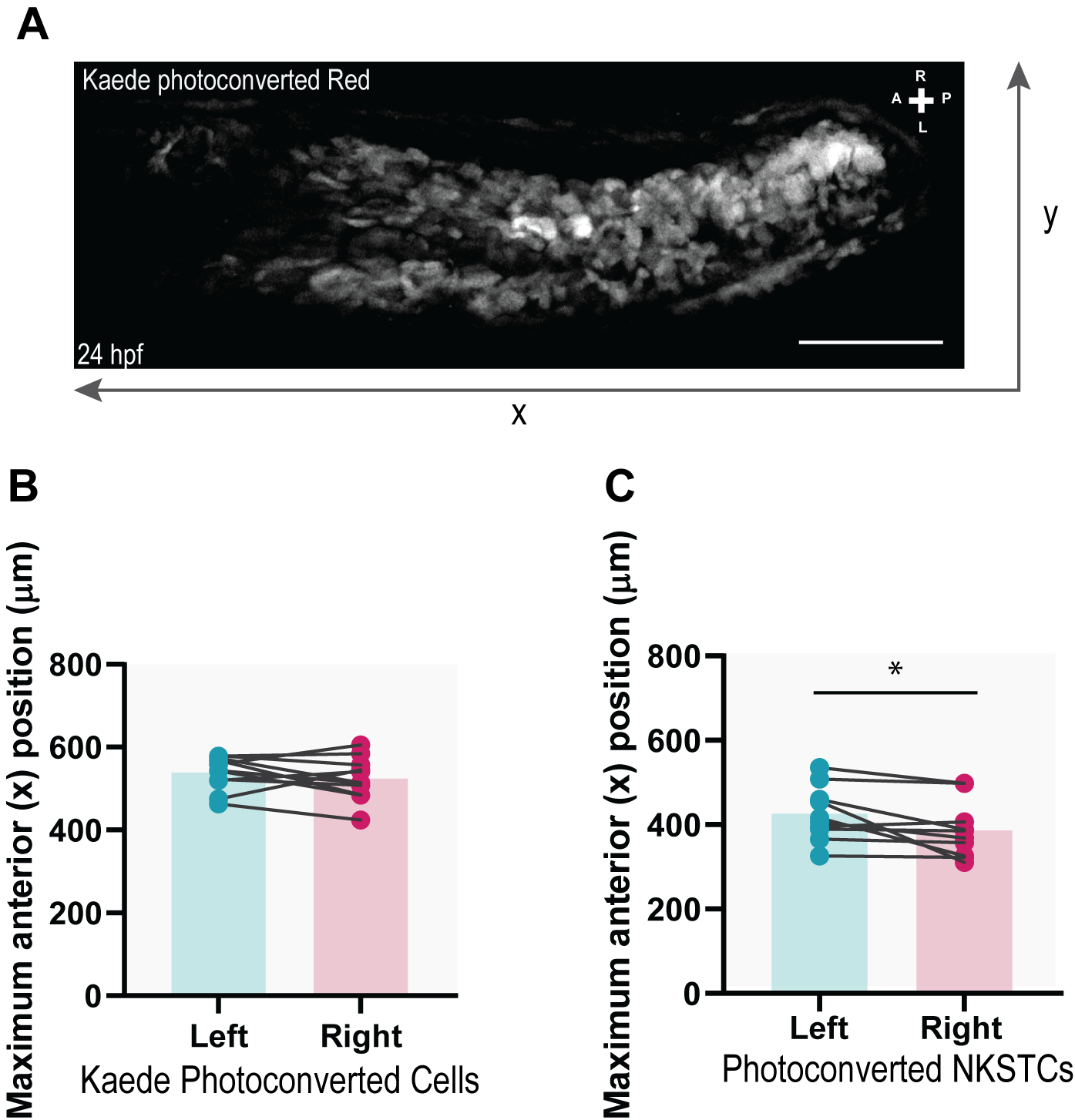

Supplement: Supplementary file 5 [file Image4.TIF]

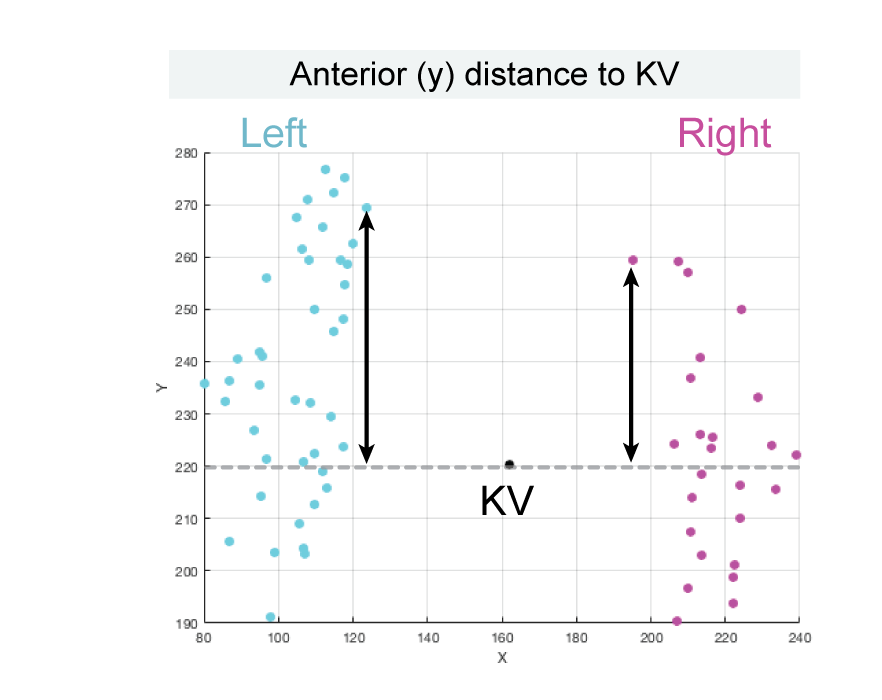

Supplement: Supplementary file 6 [file Image2.TIF]

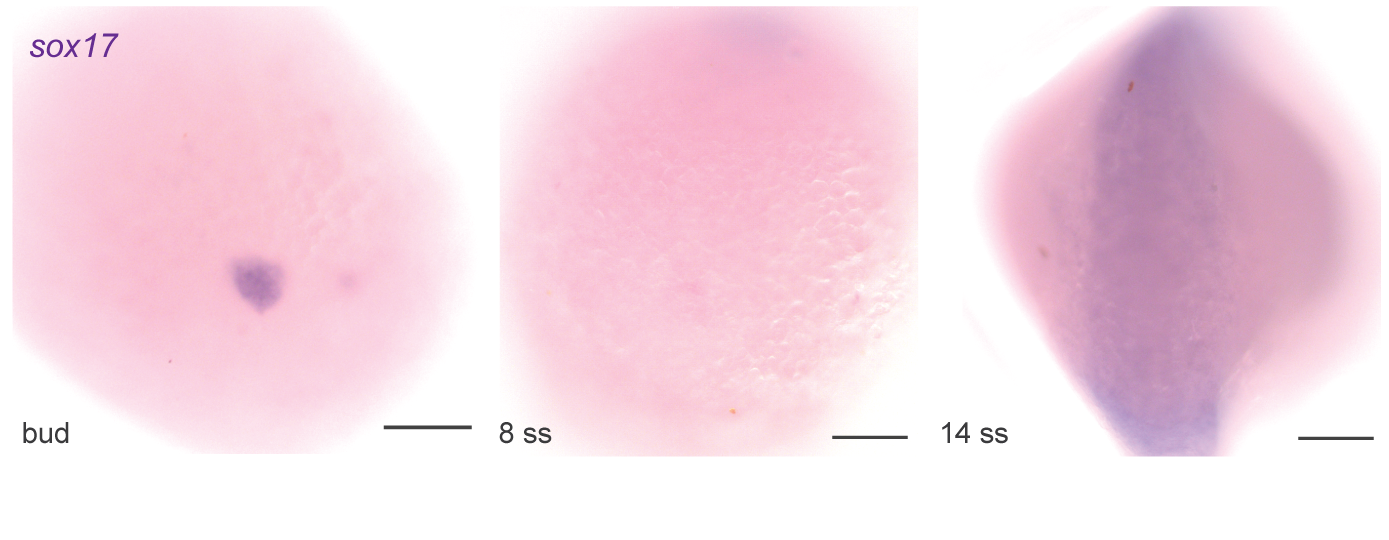

Supplement: Supplementary file 7 [file Image1.TIF]

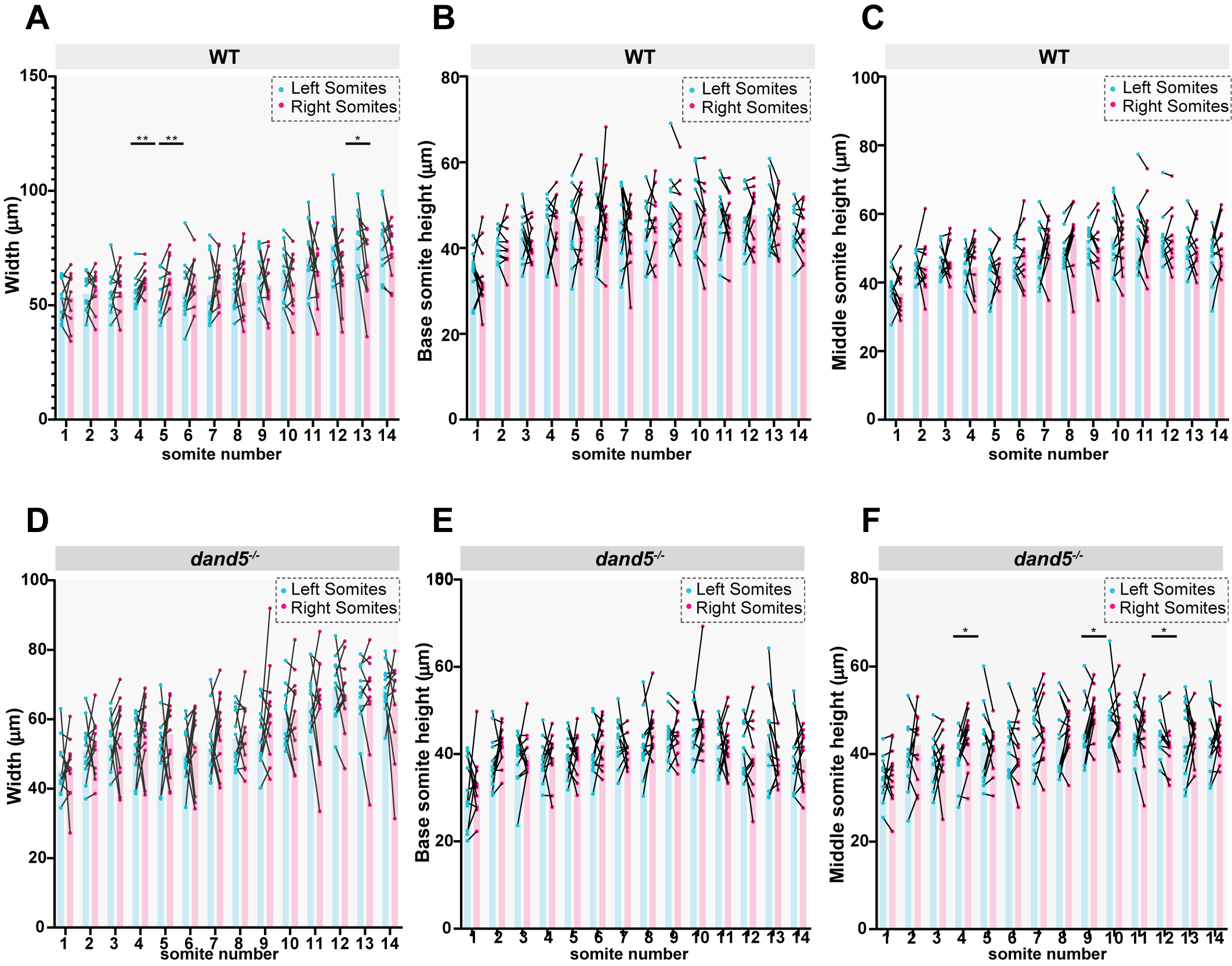

Supplement: Supplementary file 8 [file Image7.TIF]

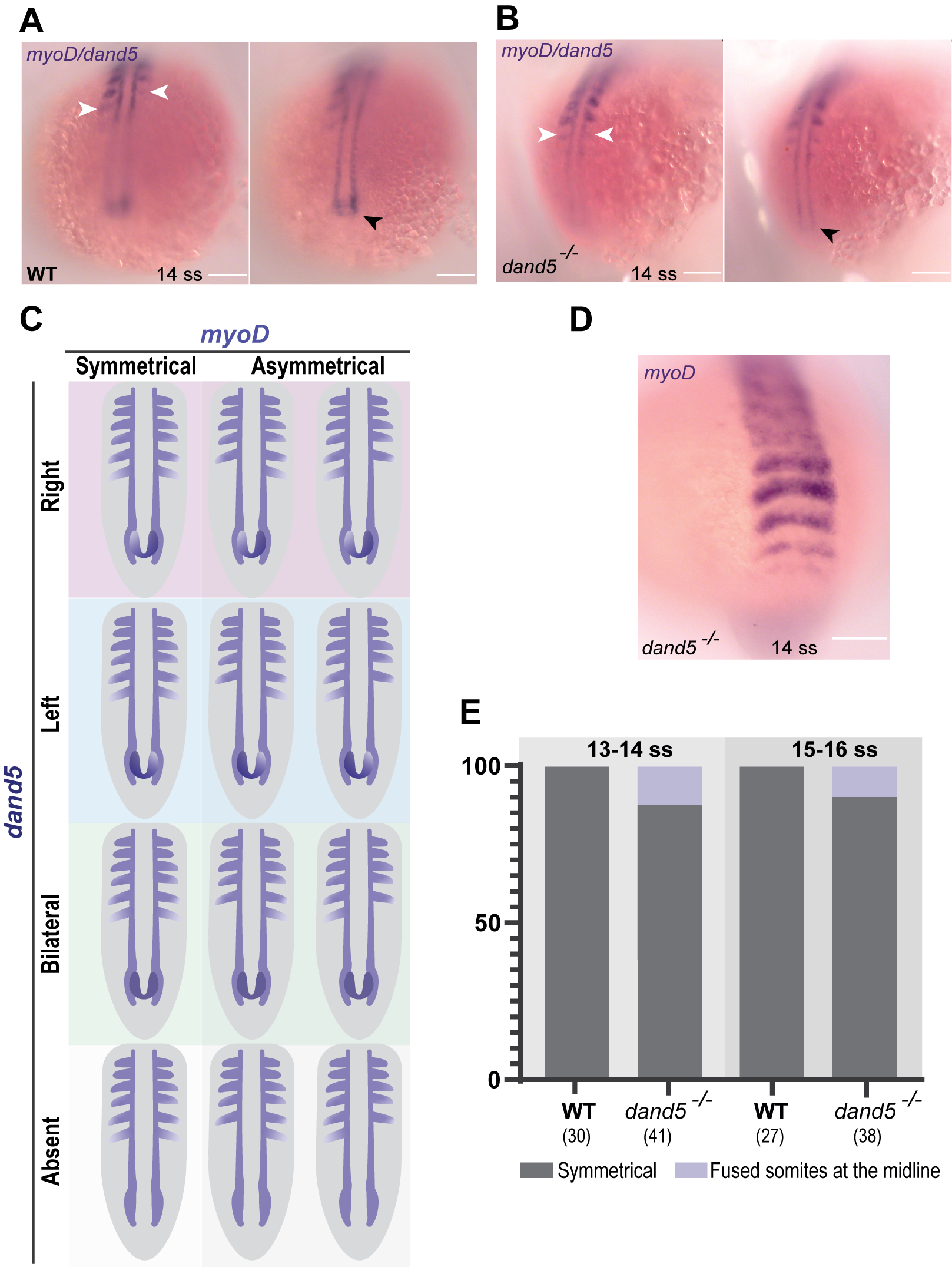

Supplement: Supplementary file 11 [file Image5.TIF]
