# Supplementary material for: Dand5 is involved in zebrafish tailbud cell movement
Source: Front Cell Dev Biol. 2023 Jan 9;10:989615. doi: 10.3389/fcell.2022.989615 (PMC9869157; doi:10.3389/fcell.2022.989615)
Supplement: Supplementary file 9 [file Table2.DOCX]

Supplementary Material

## Supplementary Figures (1-7)

## Supplementary Videos (1-5)

**Supplementary Figure 1.** sox17:GFP^+^ cells in the vicinity of the KV do not express *sox17* nor incorporate the LPM. **(A)** WISH shows that *sox17* is expressed at the bud stage in DFCs but not in the KV and cells outside the KV from 8ss onwards. Scale bar = 30 µm. Top, anterior; bottom, posterior; left, left; and right, right. **(B-C)** sox17:GFP^+^ cells did not colocalize with drl:mCherry at 18, 20, or 24 hpf, except for some gut tube cells (yellow arrowheads**).** Scale bar = 30 µm. **(B)** Lateral view of single z-planes of *T(sox17:GFP);Tg(drl:mCherry)* embryos. Left, anterior; right, posterior. **(C)** Dorsal view of single z-planes of T(sox17:GFP);Tg(drl:mCherry) embryos. The top is anterior, and the left is left.

**Supplementary Figure 2.** Schematic representation of the distance between cells and KV on the y-axis. Cyan dots and left-side cells; magenta dots: right-side cells; black dots: KV centroid.

**Supplementary Figure 3.** Effect of *dand5* absence on KV morphology. **(A)** 3D visualization of the KV in Tg(sox17:GFP) and *dand5^-/-^* raised on Tg(sox17:GFP) background embryos at 8 ss. **(B)** 3D surfaces from the KV lumen in Tg(sox17:GFP) and *dand5^-/-^* on Tg(sox17:GFP) background embryos at 8 ss. Comparison of volume **(C)**, area **(D)**, and sphericity **(E)** between WT and *dand5^-/-^* embryos. Scale bar = 500 µm

**Supplementary Figure 4.** Assessment of left-right asymmetry cell position in somites. **(A)** Maximum intensity projection of a 24 hpf embryo showing the anterior (x-axis) position of the photoconverted cells. Dorsal view: The anterior is to the right, and the left is to the bottom. Scale bar = 100 µm. **(B-C)** Quantification of the differences in the more anterior distances occupied by photoconverted cells **(B)** and NKSTCs co-labeled with photoconverted cells **(C)** per somite side. t-test paired comparisons. Bars represent mean values, and dots represent individual embryos. * corresponds to a p-value <0,05.

**Supplementary Figure 5.** *myoD* and *dand5* expression in *dand5* mutants. Double WISH for *myoD* and *dand5* expression in WT **(A)** and *dand5^-/-^* **(B)**, where *dand5* is absent. **(C)** Schematic representation of all different combinations found for the expression of both *myoD* and *dand5.* **(D-E)** Fused somites at the midline are shown by *myoD* WISH in 13-14ss and 15-16 ss *dand5^-/-^* embryos **(D)** but not in WT embryos **(E)**. Scale bar = 30 µm. The top is anterior, and the left is left.

**Supplementary Figure 6.** Morphological measurements of somites in WT and *dand5^−/−^* embryos. **(A-C)** Comparison of somite width **(A)**, base height **(B)**, and middle height **(C)** of every 14 somites in both WT and *dand5^−/−^* embryos normalized to the embryo body length.

**Supplementary Figure 7.** Comparison of morphological somite measurements between the left and right sides of the embryo. **(A-F)** Pairwise comparisons between the left and right somites of somite width in WT **(A)** and *dand5^-/-^* embryos **(D)**; somite base height of WT **(B)** and *dand5^-/-^* **(E)** and somite middle height in WT **(C)** and *dand5^-/-^* embryos **(F)** of every 14 somites. Bars represent mean values, and dots represent individual embryos. * corresponds to a p-value <0,05 and ** to a p-value <0,005.

**Supplementary Video 1.** Time-lapse imaging of asymmetric NKSTC positions from two-photon z-stacks of a Tg(sox17:GFP) embryo starting at 13 ss**.** On the left side, sox17:GFP+ cells are shown in green as a composite with transmitted light, and on the right side, in grayscale. NKSTCs reach more anterior positions on the left side of the KV. Dorsal view: The anterior is to the top, and the left is to the left.

**Supplementary Video 2.** NKSTCs migration is characterized by live imaging. Representative tracking of individual cells. Migrating cells near the KV were manually tracked in a time-lapse of two-photon z-stacks of a Tg(sox17:GFP) embryo starting at 11 ss using Imaris (Bitplane). Frames were acquired every 3 min. The tracked cells are marked by cyan and magenta spots on the left and right sides, respectively. At the starting point, spots marked the cells that started migrating at consecutive time points. The associated tracks are displayed in a heatmap color scheme for the elapsed time. Tracked cells on the left side acquired more anterior positions over time than those on the right side. Dorsal view: The anterior is to the top, and the left is to the left.

**Supplementary Video 3. An unreported cluster of NKSTCs posterior to the KV.** Time-lapse imaging from two-photon z-stacks of a Tg(sox17:GFP) embryo starting at 10 ss. Frames were acquired every 6 min. NKSTCs are present posterior to the KV. During tail elongation, the KV moves through the posterior cluster. Immediately after the KV reached the NKSTCs cluster, a few cells started leaving the left side. Soon, more cells were seen performing the same behavior on both the left and right sides, displaying asymmetric positions. Dorsal view: The anterior is to the top, and the left is to the left.

**Supplementary Video 4. KV passes through the posterior NKSTCs cluster.** 3D reconstruction from a time-lapse of two-photon z-stacks of a 10 ss *Tg*(*sox17:GFP*) embryo. The KV 3D surface (yellow) was created using Imaris (Bitplane). On the left side, the KV passes through the cluster from the dorsal view, at the center, and to the right from the left- and right-side views.

**Supplementary Video 5.** Time-lapse imaging from confocal z-stacks of a *dand5^-/-^* embryo raised on the Tg(sox17:GFP) background, starting at 10 ss. Frames were acquired every 2 min. A posterior NKSTCs cluster is present posterior to the KV. As the KV progresses posteriorly, cells leave the cluster simultaneously from the left and right sides. Dorsal view: The anterior is to the top, and the left is to the left.
